# Genetic principles enabling the creation of bacteria-based therapeutics

**DOI:** 10.1093/genetics/iyag235

**Published:** 2026-09-17

**Authors:** Ashley B Williams, Thomas M Groseclose, Sara A M Holec, Darrian Newman, Darcy S O Mora, Lyndsey Linke

**Affiliations:** SiVEC Biotechnologies, Inc, 3185 Rampart Road, Fort Collins, CO 80521, United States; SiVEC Biotechnologies, Inc, 3185 Rampart Road, Fort Collins, CO 80521, United States; SiVEC Biotechnologies, Inc, 3185 Rampart Road, Fort Collins, CO 80521, United States; SiVEC Biotechnologies, Inc, 3185 Rampart Road, Fort Collins, CO 80521, United States; SiVEC Biotechnologies, Inc, 3185 Rampart Road, Fort Collins, CO 80521, United States; SiVEC Biotechnologies, Inc, 3185 Rampart Road, Fort Collins, CO 80521, United States

**Keywords:** bacteria-based therapeutics, genetic engineering, synthetic biology, chassis selection, biocontainment, gene editing delivery, tumor microenvironment, live biotherapeutics

## Abstract

Genetic engineering can transform bacteria (including *Salmonella*, *Escherichia coli, Listeria,* and *Yersinia*) from naturally occurring microbes into programmable therapeutics. Unlike passive platforms, engineered bacteria can localize to defined biological niches, sense environmental cues, regulate programmed activity in situ, interact with host tissues in novel ways, and persist for controlled periods. These properties make bacteria versatile medicines but require genetic control of precision, safety, stability, and activity under clinical conditions. Major design layers include strain or “chassis” selection, attenuation and biocontainment, control of therapeutic activity, targeting and persistence, and genotype-to-phenotype stability during manufacturing and in vivo use. These features also create liabilities, including off-target behavior, unstable expression, and evolutionary loss of function under selection. Progress depends on safe, clinically translatable chassis with integrated, bounded, and durable behaviors. This review examines the genetic design principles and liabilities of bacteria-based therapeutics, approaches used to produce clinical and near-clinical candidates, and the emergence of programmed bacteria delivering complete gene editing systems to therapeutically relevant cells.

## Introduction

Recognition that conventional treatment modalities cannot fully address many diseases is reshaping therapeutics development (Choi et al. 2023; Lu et al. 2023; Sharma et al. 2024). Small molecules and other traditional modalities have transformed medicine but remain limited in complex, heterogeneous, or intractable diseases. Many targets and pathways remain inaccessible, on-target specificity can be constrained, resistance can emerge, and durable control can be difficult (Coleman and Rodon 2021; Lu et al. 2023; Xie et al. 2023). Accordingly, interest is growing in strategies that expand the range of tractable targets, enable new mechanisms, and support precise intervention. This shift is driving next-generation modalities intended to overcome conventional drug constraints where unmet need remains high (Meyer et al. 2021; Sankar et al. 2024; Baek et al. 2025). These next-generation modalities, including antibodies (Sharma et al. 2023; Paul et al. 2024; Strohl 2025), cytokines (Rubahamya et al. 2024), nucleic acid therapeutics (Kulkarni et al. 2021; Parhiz et al. 2024; Makkar 2025; Naeem et al. 2025), gene-editing systems (Doudna 2020; Geng et al. 2025; Liu et al. 2025), and advanced delivery platforms (Mora et al. 2023; Guo et al. 2024; Bian et al. 2025; Schwarz et al. 2025; Uzakova et al. 2025), offer broad specificity and functional scope but introduce new clinical translation demands (Dirisala et al. 2023; Balash et al. 2025). Success depends on both molecular mechanism and controlled, safe, reproducible delivery to the correct tissue, cell type, or intracellular compartment, together with cost-effective and reproducible manufacturability. As therapeutic modalities (Zhou et al. 2018) become more sophisticated, their promise increasingly depends on precise, context-dependent activity in patients.

Recombinant DNA technologies and synthetic biology advances (Agrawal et al. 2025; Qiao et al. 2026) enable bacteria as programmable therapeutic systems (Forbes et al. 2018; Raman et al. 2023; Manole et al. 2025). This progress has driven interest in bacterial therapeutics as a living modality distinct from nonliving drugs. Intrinsic and engineered functions allow bacteria to localize to specific tissues, including tumors, sense local conditions, produce therapeutic effectors in situ, and persist long enough for sustained activity. Their behavior can therefore be coupled to the disease environment. Unlike systemically delivered drugs, engineered bacteria can integrate localization, sensing, production, and release within one system, enabling conditional action in defined pathological contexts (Forbes et al. 2018; Raman et al. 2023). The same features that make bacteria therapeutically attractive also complicate translation and require careful control (Raman et al. 2023; Liu et al. 2026). Growth, adaptation, persistence, and direct interactions with host physiology, microbiota, and tissues create challenges beyond payload choice or administration route. A useful therapeutic must reach its target with controlled biodistribution, persistence, and activity and an acceptable safety profile. Translation therefore requires precise, safe, and reproducible control in vivo, not efficacy in simple preclinical models alone.

Bacteria have been considered and tested therapeutically for more than a century (Busch 1868; McCarthy 2006). The central question is now whether genetic engineering can harness bacterial biology to produce regulatable therapeutic systems. The strongest candidates will be distinguished not only by their payloads but by how their underlying biology supports defined therapeutic behavior. Strain identity, genome stability, safety, targeting, persistence, host interaction, and activity control are therefore core design features. Genetic engineering must be viewed as both discovery and manufacturing tools as well as a means of constructing a clinically usable bacterial therapeutic. Natural and engineered bacterial therapeutics differ fundamentally. Probiotic and other non-engineered strains are used for existing properties, e.g. ecological compatibility, host association, or beneficial local effects (Sarita et al. 2024; Cho et al. 2025). By contrast, engineered therapeutics use a starting “chassis” strain whose localization, environmental sensing, payload production, host interaction, persistence, and activity can be genetically specified and controlled. Bacteria-based therapeutics require interdependent design layers, beginning with chassis selection and followed by modifications that create a safe and effective system (Fig. 1). Chassis selection shapes genetic tractability, manufacturability, and host compatibility. Subsequent engineering must encode therapeutic moieties and optimize biodistribution, target access, and persistence (i.e. bacterial elimination after activity or continued replication in the patient). Genetic engineering therefore defines the pharmacological features of a bacteria-based therapeutic. Importantly, regulators increasingly recognize that these systems cannot be evaluated using the pharmacokinetic and pharmacological assumptions applied to traditional drug classes such as small molecules (Paquet et al. 2021; Pan et al. 2025). This review examines how these design layers combine in next-generation bacteria-based therapeutics, emphasizing current clinical and near-clinical assets.

**Fig. 1.**
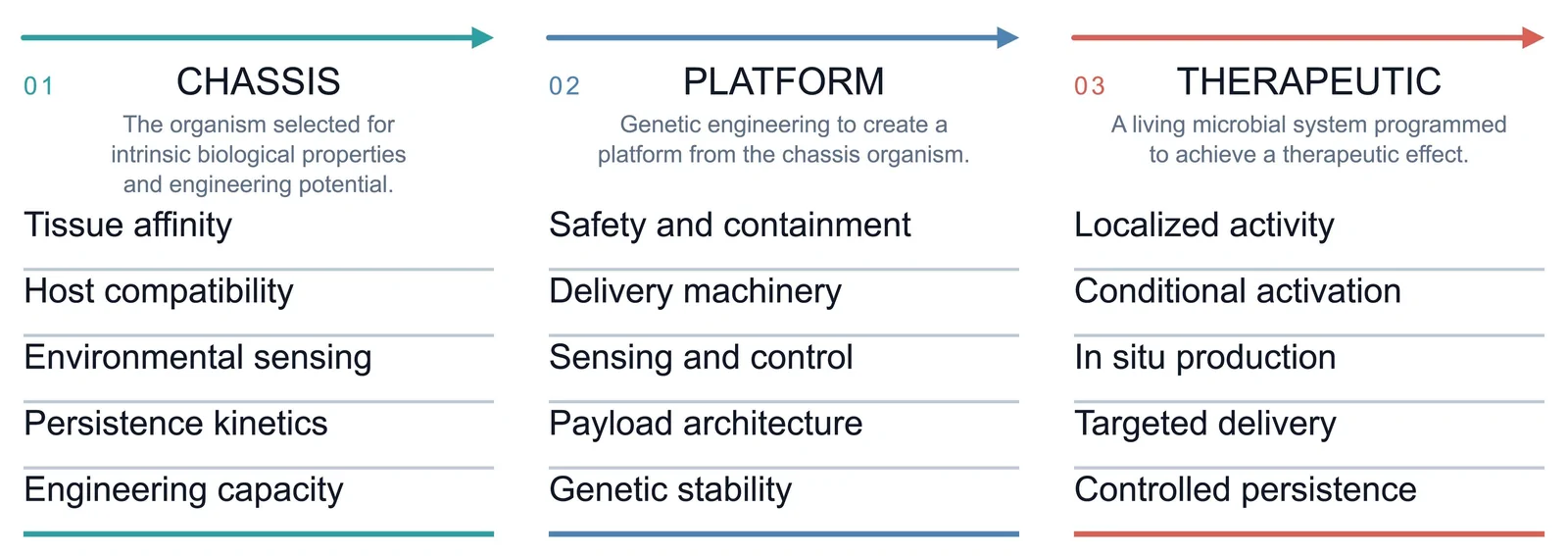
Conceptual framework linking chassis selection, platform engineering, and therapeutic function in engineered bacterial therapeutics. Native bacterial organisms are first selected as chassis based on intrinsic biological properties, including tissue affinity, host compatibility, environmental sensing, persistence behavior, and engineering tractability. Genetic engineering then converts the chassis into a deployable platform through the introduction of programmable safety systems, delivery machinery, sensing and control circuits, payload architectures, and genetic stability features. Finally, therapeutic configuration programs the engineered bacterium to achieve a defined therapeutic function, including localized activity, conditional activation, in situ therapeutic production, targeted delivery, and controlled persistence.

### Genetic and physiological considerations in chassis selection

A chassis must be selected before payload design, attenuation, or containment. Its intrinsic features influence biodistribution, host interactions, tolerance for genetic burden (e.g. plasmid size or protein expression), and cost-effective, reproducible manufacturing (Mu and Zhang 2023). Thus, chassis serves as the biological and genetic scaffold for application-specific design. These same properties constrain engineering, so various therapeutic contexts require different strains to accommodate specific payload expression, persistence, tissue access, host compatibility, and manufacturing reproducibility. A strain suited to durable gastrointestinal colonization might be ineffective systemically, whereas a tumor-localizing strain might be unsuitable for broad biodistribution or repeated administration. Chassis selection must therefore match microbial traits and engineerable features to the therapeutic problem. The principal chassis classes are probiotic strains, native or commensal strains, and attenuated pathogens, which differ in physiology and engineering tractability (Table 1).

**Table 1.** Common chassis classes used in bacteria-based therapeutics.

Chassis class	Basis for selection	Main advantage	Main limitation	Representative examples
Probiotic strains	Host-compatible, engineerable backgrounds	Favorable tolerability and established tools	Limited intrinsic access to some disease sites	*E. coli* Nissle 1917; *L. lactis, L. reuteri*
Native or commensal strains	Natural adaptation to the target niche	Strong ecological fit	Historically limited genetic tractability	*S. epidermidis*
Laboratory strains	Deeply characterized, highly engineerable backgrounds	High design flexibility	Limited native therapeutic fitness	*E. coli* K-12/MM294
Attenuated pathogens	Native host-interaction traits repurposed through attenuation	Intrinsic invasion, intracellular delivery, or tissue colonization	Greater safety and regulatory liabilities	*S. enterica* Typhimurium*, Y. enterocolitica, L. monocytogenes*

Representative bacterial chassis classes, their basis for selection, principal advantages and limitations, and illustrative examples relevant to therapeutic development.

Probiotic strains offer favorable, well-understood host compatibility, a history of human use, and sometimes regulatory familiarity. *E. coli* Nissle 1917 (EcN) is a well-known example that has been approved by the US Food and Drug Administration (FDA) for several clinical trials (for example, see NCT02802059, NCT01696227, NCT02276508). Synlogic, a clinical-stage biopharmaceutical company, has leveraged EcN in its therapeutics pipeline. In its SYNB1891 program, EcN was engineered to produce cyclic di-AMP as an intratumoral STING agonist, coupling the tractability of the EcN chassis with controlled local immune activation in the TME. EcN remains attractive because its host-compatibility profile and genetic toolbox support applications from gastrointestinal delivery to tumor-localized immunomodulation. Aurealis Therapeutics has advanced its bacteria-based therapeutic, built on a strain of the well-known probiotic *Lactococcus lactis*, *Lactococcus cremoris* (*Lactococcus lactis* subsp. cremoris) (Song et al. 2017; Darby et al. 2019), into clinical evaluation. Their lead asset, AUP-16, leverages this chassis as a topical in situ production system for FGF-2, IL-4, and CSF-1 for the treatment of ulcerative diseases.

Host-associated commensals can be harder to engineer because their toolsets are less established. Azitra's therapeutic pipeline builds in part on the academic work of Oh and collaborators, who have shown that skin-associated *Staphylococcus epidermidis* strains encompass extensive genetic and phenotypic diversity that afford therapeutic potential (Zhou et al. 2020). In this framework, *S. epidermidis* is attractive as a human skin commensal that is highly adapted to the cutaneous environment; however, successful development of *S. epidermidis-*based therapeutics requires overcoming limited genetic tractability. Azitra's system clones a therapeutic gene into a platform plasmid or integrates it into the chromosome of a specific *S. epidermidis* strain. This approach generated the topical therapeutics ATR-12, ATR-04, and ATR-01.

Laboratory strains provide another chassis strategy. *E. coli* K-12 derivatives are especially attractive because they offer deep genetic annotation, extensive biological knowledge, reliable growth, and mature tools for plasmid manipulation, chromosomal editing, controlled expression, and manufacturing. Cequent Pharmaceuticals’ (now inactive) CEQ508 therapeutic (D’Cruz et al. 2017; Trieu et al. 2017) was built on *E. coli* K-12 derivative MM294 (Meselson and Yuan 1968). To create CEQ508, MM294 was modified for mucosal delivery of therapeutic short hairpin RNA (shRNA) via trans-kingdom RNA interference (Nguyen and Fruehauf 2009). CEQ508 used a tractable strain with a broadly accepted safety profile rather than a probiotic or commensal selected for ecological compatibility. The FDA granted CEQ508 Fast Track status (Healio 2015).

Attenuated pathogens offer a different set of potential therapeutic chassis. For example, attenuated *Salmonella* strains retain traits supporting tissue penetration, tumor colonization, and host-cell interaction, which can enable delivery to poorly perfused or immunologically distinct regions (Becerra-Báez et al. 2022; Agrawal et al. 2025). However, they carry greater translational and regulatory concerns about attenuation, dissemination, inflammatory burden, residual virulence, and attenuation stability (discussed in detail in the liabilities section below). These principles are evident in some of the most advanced *Salmonella*-based oncology programs. Actym Therapeutics, a clinical-stage biotechnology company developing bacterial immunotherapies for cancer, illustrates how attenuated *Salmonella enterica* serovar Typhimurium can be converted into a modular therapeutic platform via programmed payload expression within the TME. In its STACT platform (Cron et al. 2025), the natural tumor homing behavior of the *Salmonella* chassis can be combined with a genetically programmed therapeutic cargo. The former restricts biological activity to the disease site, thereby reducing systemic toxicity, while the latter provides flexibility in therapeutic development. Actym's lead program, ACTM-838 (Cron et al. 2025), exemplifies how the clinical viability of attenuated *Salmonella* depends on this integration of chassis behavior with an engineered fit-for-purpose payload. A complementary example, also built on *S. enterica* serovar Typhimurium, is provided by the work of Ernest Pharmaceuticals, whose BacID platform demonstrates how an attenuated *Salmonella* chassis can be engineered into an intracellular delivery system rather than exploited solely for its tumor-localizing properties (Raman et al. 2021). The engineering challenge is to coordinate tumor localization, tumor-cell invasion, intracellular residence, and cargo release rather than retain pathogenesis-associated traits. The chassis combines tumor localization with sufficient tractability for this reprogramming. Ernest's lead candidate, EBT-002, couples this platform to a defined payload.

T3 Pharma (now part of Boehringer Ingelheim) developed a pathogen-derived delivery vehicle from a *Yersinia enterocolitica* chassis (Ittig et al. 2015). The therapeutic value of attenuated *Y. enterocolitica* combines natural tumor tropism with a repurposable type 3 secretion system (T3SS). T3 Pharmaceuticals’ lead candidate, T3P-Y058-739, entered a phase 1/2 trial (see NCT05120596) for advanced solid tumors, demonstrating clinical translation despite the liabilities of a pathogenic chassis.

Importantly, these examples show how the same chassis can be used as a multiproduct platform: Synlogic has used EcN to address multiple rare metabolic diseases by engineering distinct metabolic pathways (e.g. SYNB1934 and SYNB1353). Similarly, Azitra has leveraged both concepts: they have assembled a microbiome-derived library of *S. epidermidis* strains with useful intrinsic features and engineered them to secrete human proteins for ATR-12, ATR-04, and ATR-01. Aurealis has used a *Lactococcus* probiotic chassis for wound healing (AUP-16), oncology (AUP-55), and inflammation (AUP-18 and AUP-20). Likewise, Actym and Ernest Pharmaceuticals used intrinsic *Salmonella* to produce and deliver proteins or oncolytic viruses (ACTM-838 and undisclosed assets; EBT-002, EBT-407, and EBT-305). T3 Pharmaceuticals expanded its *Y. enterocolitica-*based pipeline with T3SS-delivered cargos (T3P-Y058-739, T3P-Y098, T3P-1017, and T3P-1127).

### Seminal studies on cargo production and delivery by bacteria

A bacterial therapeutic becomes meaningfully distinct from the starting strain when therapeutic moiety production and clinically relevant non-intrinsic behaviors are engineered into the chassis (see Table 2 for examples). Performance depends on cargo identity, expression level and duration, and coupling to bacterial localization, persistence, and survival. Seminal studies of bacteria-mediated delivery first established these principles.

**Table 2.** Conserved chassis-engineering features across bacteria-based therapeutic platforms.

Platform (sponsor)	Chassis	Chassis engineering	Biocontainment	Staged control	Context-responsive regulation
BM2710 (Courvalin)	*E. coli* K-12/MM294	+invasion functions (pathogen-derived)	Δ*dapA* (DAP aux.); abortive survival	receptor-mediated targeted entry/death	LLO-gated release (acidified phagosome)
CEQ508 (Cequent)	*E. coli* K-12 CEQ221	plasmid *inv + hlyA;* Δ*dapA*, Δ*rnc* (RNA delivery)	Δ*dapA*; intracellular death	receptor-mediated targeted delivery/shRNA	LLO-gated release (acidified phagosome)
BactPac (SiVEC)	*E. coli* K-12 SVC1	chr. *inv* + *hlyA*	Δ*dapA*	receptor-mediated targeted delivery/payload	LLO-gated release (acidified phagosome)
T3P (T3 Pharma)	*Yersinia* *enterocolitica*	ΔYop, +SycE (T3SS cargo)	Δ*asd* (DAP aux.)	T3SS/cargo	—
BacID (Ernest)	*S. enterica* Typhimurium VNP20009	rewired motility/invasion; Δ*sseJ* (vacuolar)	Δ*asd* + *asd*^+^ plasmid; programmed lysis	colonize-invade- reside-lyse	P*sal*–*flhDC* (salicylate); P*sseJ*–*lysE* (vacuole)
STACT (Actym)	*S. enterica* Typhimurium VNP20009	ΔmsbB, *pagP, fljB, fliC, csgD, ansB, purI*	Δ*asd* + *asd*^+^ plasmid	deliver-enrich- uptake-lyse	chassis-intrinsic (no circuit)
A1-R (Zhao/Hoffman)	*S. enterica* Typhimurium	Chemical mutagenesis for Leu/Arg auxotrophy; tumor passage and re-isolation	Leu/Arg auxotrophy restricts growth in normal tissues	—	– (passive nutrient-dependent tumor selectivity)
Saltikva (Salspera)	*S. enterica* Typhimurium	Δ*cya*, Δ*crp*; pIL2 (truncated hIL-2)	Δ*asd* + *asd*^+^ plasmid (antibiotic-free)	colonize- IL-2 release	intrinsic tropism (no promoter)
SYNB1618 (Synlogic)	*E. coli* Nissle 1917	Phe-degradation pathway	Δ*dapA*	*mfg* induction/ gut activity	P*fnrS* (anaerobic gut); P*tac*/PBAD (mfg)
MMD (Matter Bio)	*L. monocytogenes* XFL-7	Δ*prfA* + partial *prfA*; LLO–antigen fusion	Δ*prfA* (avirulent); nonhemolytic LLO	accumulate-infect- escape-present-recall	tumor-colonization coupled (no promoter)
AUP (Aurealis)	*L. lactis cremoris* AUC1000	Δ*alr*; Usp45 secretion, single multi-cargo operon	Δ*alr* (antibiotic-free); no kill switch reported	secretion/delivery; one operon	P*gadC*/*gadCB* + GadR (chloride)
ILP100 (Ilya)	*L. reuteri* R2LC	+CXCL12-1α expression construct	None engineered; transient persistence without engraftment	SppIP activation/CXCL12 production and local delivery	—

Representative platforms compared by core chassis-level design features: chassis engineering, biocontainment, staged genetic control, and context-responsive regulation. Gene symbols are italicized; protein names are in roman type. Symbols: Δ, deletion; +, added function;/, functionally separated; -, staged sequence. Abbreviations: Aux., auxotrophy; chr., chromosomal; DAP, diaminopimelic acid; hIL-2, human interleukin-2; IL-2, interleukin-2; LLO, listeriolysin O; LPS, lipopolysaccharide; mfg, manufacturing; Phe, phenylalanine; shRNA, short hairpin RNA; T3SS, type III secretion system; trunc., truncated.

In a 1995 study, Courvalin and colleagues (Courvalin et al. 1995) used classical genetic engineering to convert a tractable nonpathogenic strain into a delivery vehicle using pathogen-derived genes. Earlier unpublished work showed that a *dapB* mutant of *Shigella flexneri* M90T, which is auxotrophic for diaminopimelic acid (DAP), could deliver replicative or integrative vectors intracellularly in HeLa and COS cells after a 1-hour co-incubation; importantly, transfer required cell invasion and phagosomal escape encoded on the *S. flexneri* virulence plasmid pWR100. They therefore transferred these functions from a pathogen into a well-understood nonpathogenic strain, selecting a laboratory *E.* coli K-12 derivative. They constructed their first delivery strain, BM2710, by assembling chromosomal alleles from Matt Meselson's MM294 strain. During this engineering stage, they also introduced an insertional deletion of *dapA* to recapitulate the DAP auxotrophy of *S. flexneri.* They then introduced a derivative of the pWR110 plasmid carrying a kanamycin resistance cassette (pWR100::Tn*5*) into this intermediate strain to confer invasiveness to mammalian cells. Finally, they transformed the resulting strain with two plasmids encoding test cargo: one coded for β-galactosidase expression in the recipient mammalian cells and one conferred hygromycin resistance in the mammalian cells upon successful delivery. In A549 and CHO “transcipients” (i.e. the mammalian cells that were invaded by the bacteria), they showed that the plasmids were intact and replicated, while the number of viable intracellular BM2710/pWR110 cells declined sharply over 24 to 72 h, consistent with the intended abortive invasion design conferred by the *dapA* deletion. This study established the core architecture that would serve as a paradigm for future advances in bacteria-based cargo delivery, i.e. invasion supplied by exogenous pathogen-derived genes and auxotrophy-induced clearance.

In key follow-up work by Grillot-Courvalin and colleagues (Grillot-Courvalin et al. 1998), this design separated invasion from phagosomal escape in a more modular platform. Starting with BM2710, they replaced reliance on the large, complex *Shigella* virulence plasmid with two synthetic plasmids: pGB2Ωinv, which carried the *Yersinia pseudotuberculosis inv* gene (encoding invasin), and pGB2Ω-*hly*, which encoded the *Listeria monocytogenes hlyA* gene (encoding listeriolysin O, LLO). Importantly, LLO is only activated at low pH (<6) (Schuerch et al. 2005), rendering it inactive until the bacteria have been internalized in phagosomes, which are acidified by the target cell. The restricted activity of LLO mitigates collateral damage to host cell membranes. Phagosomal disruption equilibrates the pH with the cytoplasm, inactivating LLO and limiting toxicity. This architecture separates bacterial entry into non-phagocytic β1-integrin-positive target cells via the invasin protein from LLO-mediated phagosomal escape. For clinical translation, they also demonstrated delivery to nondividing cells. The partitioning of target-cell entry from cargo deposition remains central to modern bacteria-based delivery platforms.

By 2004, the key question in the field had shifted from whether a genetically programmed *E. coli* strain could deliver cargo to whether it could reach clinically relevant cells. Fajac and colleagues (Fajac et al. 2004) continued working with BM2710 now programmed with a consolidated version of the same delivery logic. Bacteria carrying the plasmid pGB2Ω*inv-hly* (with *inv* and *hlyA* now encoded on a single plasmid, thereby reducing plasmid burden and increasing flexibility) could deliver DNA cargo to immortalized airway epithelial lines, including cystic fibrosis-derived cells. Castagliuolo and colleagues (Castagliuolo et al. 2005) then reconceptualized the same *E. coli* platform (BM2710 pGB2Ω*inv-hly*) from proof of concept to a colon-directed therapeutic system. The first payload was pC1ΩTGF-β1, a eukaryotic expression plasmid that allowed the target cells to express TGF-β1 from a constitutive promoter. They first demonstrated that TGF-β1 mRNA was present in CMT-93 colonic epithelial cells treated with BM2710/pGB2Ω*inv-hly* carrying pC1ΩTGF-β1. Remarkably, oral delivery of this strain reduced disease severity in a mouse colitis model; however, mucosal injury eliminated spatial restriction. In colitic animals, plasmid-specific transcripts appeared in the colon and extra-colonic tissues because of epithelial barrier disruption, showing that intended bacterial biodistribution might not hold in vivo. The authors therefore replaced the constitutive promoter with the human IL-8 promoter, which is responsive to inflamed conditions; thus, cargo expression was linked to inflammation at the intended site (the mouse colon). The inducible construct confined expression to the target site, preserving efficacy while limiting off-target effects. This advance retained bacterial transport and cargo deposition while encoding disease-state specificity. It also raised a continuing design question: when is cargo-level restriction sufficient, and when must the vehicle encode spatial control? The Castagliuolo data suggest that chassis-level biodistribution alone cannot be relied upon when barrier integrity is compromised, a caveat for any setting in which the target tissue is also actively diseased. The field has increasingly shifted spatial control from cargo to vehicle, although the optimal combination remains disease-specific.

Xiang and colleagues (Xiang et al. 2009) then implemented a paradigm shift in the system by transitioning cargo production from the mammalian cells to the bacterial delivery vehicle. Their trans-kingdom RNA interference (tkRNAi) system used a redesigned architecture in which a single plasmid carried the invasion and phagosomal escape functions (*inv* and *hlyA*) as well as an shRNA expression cassette under the control of a T7 promoter. This plasmid, which encoded both the core delivery functions and the cargo, was introduced into BL21(DE3), which supplied the T7 RNA polymerase for shRNA synthesis inside the bacterium. This design represented a clear mechanistic departure from earlier studies and showed that therapeutic bacteria can produce and deliver cargo. Xiang also confirmed delivery of biologically active bacterially produced cargo to target cells.

These early studies reduced the genetic requirements for bacterial cargo production and delivery to a reusable module. More recent platforms encode attenuation, tissue tropism, host interaction, payload retention, and deployment in the chassis, allowing rapid substitution of cargos (Fig. 2). The following sections summarize selected clinical and near-clinical efforts built on these concepts (Table 3).

**Fig. 2.**
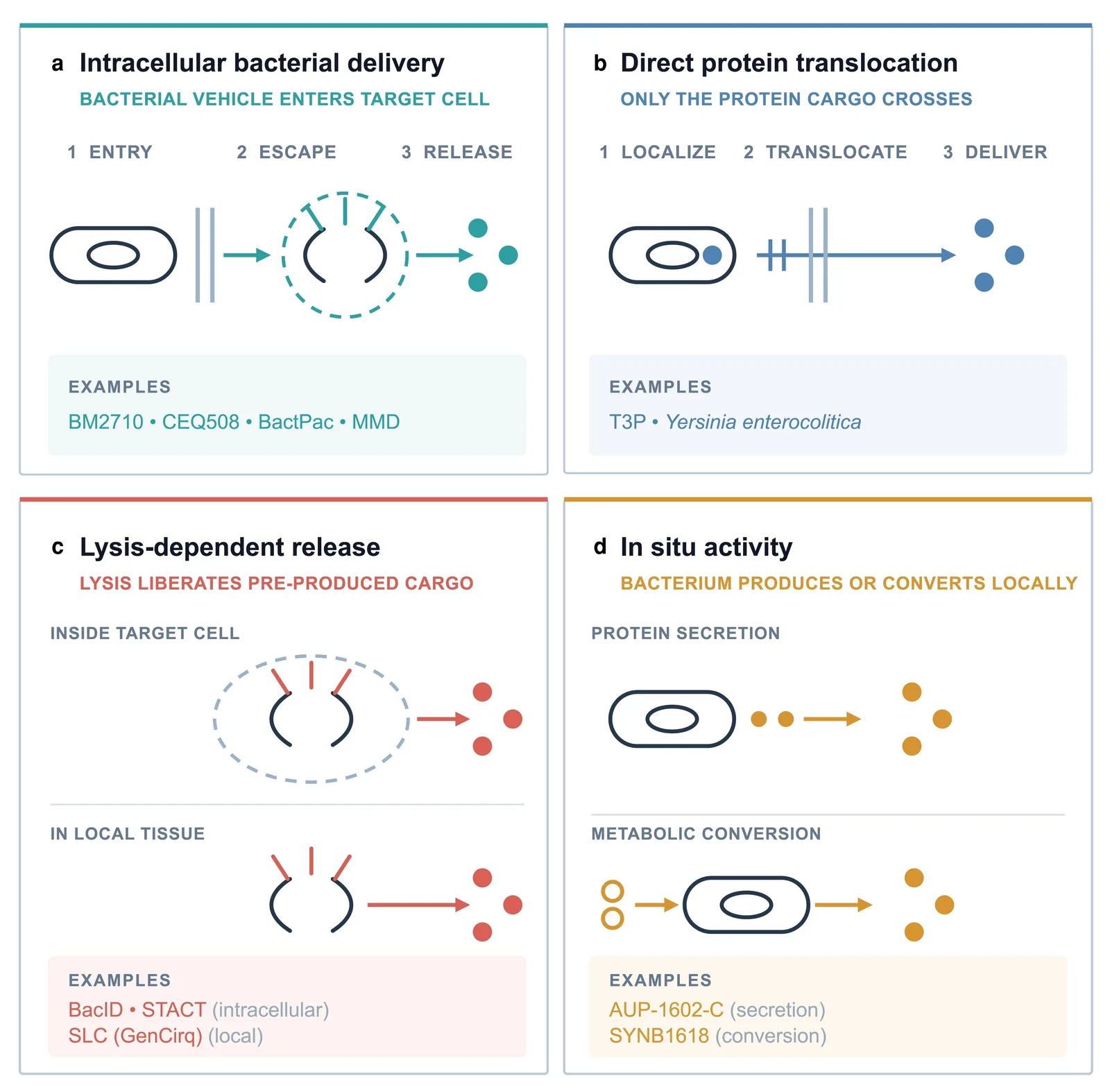
Therapeutic production and deployment architectures in engineered bacterial therapeutics. a) Intracellular bacterial delivery involves entry of the bacterial vehicle into a target cell, followed by bacterial lysis and cargo escape from the host vacuole or phagosome, as illustrated by BM2710, CEQ508, BactPac, and MMD. b) Direct protein translocation allows extracellular bacteria to inject protein cargo across the host-cell membrane without bacterial entry, as exemplified by T3P and engineered *Y. enterocolitica*. c) Lysis-dependent architectures release pre-produced cargo through bacterial rupture, either within target cells, as in BacID and STACT, or in local tissue, as in the synchronized lysis circuit developed by GenCirq. d) In situ activity maintains intact, metabolically active bacteria at the treatment site, enabling either protein secretion, as in AUP-1602-C, or substrate uptake and metabolic conversion, as in SYNB1618. These architectures differ in the location of the bacterial vehicle, the form of the therapeutic cargo, and the mechanism and timing of cargo deployment.

**Table 3.** Selected clinical trials of engineered bacterial therapeutics.

Program/sponsor	Chassis organism	Indication	Trial/phase	Status	Enrol.	Start	Primary completion
Saltikva (Salmonella-IL2) Salspera LLC	*S. enterica*	Metastatic pancreatic cancer	NCT04589234 Phase 2	Active, not recruiting	60	2020-10	2030-12 (est.)
ZH9 (PARADIGM-1) Prokarium Ltd	*S. enterica*	High-risk non-muscle-invasive bladder cancer	NCT06181266 Phase 1/1b	Active, not recruiting	22	2024-01	2025-08
T3P-Y058-739 T3 Pharmaceuticals	*Y. enterocolitica*	Advanced solid tumors (+ pembrolizumab)	NCT05120596 Phase 1/2	Recruiting	100	2022-04	2027-02 (est.)
ATR12-351 Azitra Inc.	*S. epidermidis*	Netherton syndrome (topical)	NCT06137157 Phase 1	Recruiting	12	2024-06	2026-06 (est.)
ATR04-484 Azitra Inc.	*S. epidermidis*	EGFR-inhibitor-associated dermal toxicity	NCT06830863 Phase 1/2	Recruiting	32	2025-08	2027-01 (est.)
LM-LLO-TT Matter Bio	*L. monocytogenes*	Pancreatic ductal adenocarcinoma	Pending Phase 1/2a	IND-approved	Pending	Pending	Pending
Clostridium novyi-NT + pembrolizumab Biomed Valley Discoveries	*Clostridium novyi*	Treatment-refractory solid tumors	NCT03435952* Phase 1b	Active, not recruiting	18	2018-07	2026-10 (est.)
SGN1 (intravenous) Guangzhou Sinogen	*S. enterica*	Advanced solid tumors	NCT05038150* Phase 1/2a	Recruiting	70	2023-01	2028-01 (est.)
SGN1 (intratumoural) Guangzhou Sinogen	*S. enterica*	Advanced solid tumors	NCT05103345* Phase 1/2a	Recruiting	70	2023-04	2027-12 (est.)
ILP100-Topical Ilya Pharma AB	*Limosilactobacillus reuteri*	Wound healing	EudraCT 2019-000680-24 Phase 1 (Öhnstedt et al. 2023)	Completed (Successful safety outcomes and wound healing)	36	2019-09	2021-10

Estimated dates are indicated by “est.” Trials marked with an asterisk (*) involve assets not otherwise covered in this review; the cited references are provided for further information.

### *Escherichia coli-*based therapeutics

*E. coli* is a major therapeutic chassis because its genetic tractability supports diverse strategies. Across probiotic, laboratory-derived, and application-optimized strains, *E. coli* has been engineered for local production, nucleic acid transfer, and intracellular delivery. This section highlights *E. coli*-based nucleic acid delivery, while additional *E. coli* systems appear below where they illustrate other expression and delivery strategies.

An early clinically advanced example was CEQ508 from Cequent Pharmaceuticals, in which a genetically modified *E. coli* chassis was configured for oral delivery of RNA interference cargo to the gastrointestinal (GI) tract. CEQ508 commercialized the technologies described above through stepwise modification of MM294. The platform strain CEQ221 carried deletions of *dapA,* which supported maximized cargo delivery and biocontainment, and *rnc,* which encodes RNase III (to stabilize RNA cargo). The latter was motivated by the intended purpose of CEQ221, i.e. to support the development of multiple RNA-based therapeutics. CEQ508 added a plasmid encoding invasion (*inv*), phagosomal escape (*hlyA*), and therapeutic shRNA production. The shRNA was designed to reduce β-catenin levels via tkRNAi in target cells to treat familial adenomatous polyposis (FAP). CEQ508 demonstrated that an innovative therapeutic could be derived from a common *E. coli* laboratory strain using standard genetic techniques of the time (primarily chromosome modification via the method of Datsenko and Wanner) (Datsenko and Wanner 2000). CEQ508 gained fast-track designation from the FDA and was successful in phase I safety trials (D’Cruz et al. 2017; Trieu et al. 2017) when administered orally to reach the GI tract, which was compatible with an intrinsic feature of the *E. coli* chassis. Despite its early success, the status of the CEQ508 program is unclear at this time.

As described in a 2023 paper (Mora et al. 2023), SiVEC Biotechnologies, Inc., developed the SVC1 platform strain. Importantly, SVC1 and its later derivatives (see BactPac below) specifically target the cell surface marker CD29 (via invasin), enabling cell targeting, and then enter the cells where they lyse, resulting in phagosomal perforation (via LLO) to allow cargo release into the cytoplasm. The SVC1 platform was first used in SiVEC-IAV, which carries a plasmid-borne expression cassette encoding two shRNAs targeting the influenza A virus (IAV) NP and PA genes from a genetic design standpoint, the use of shRNAs allows compact payload cassettes to be maintained in the bacterial vector and processed by mammalian RNA interference machinery after delivery (Linke et al. 2016). After delivery, shRNAs form stem-loop precursors that the canonical RNAi pathway processes into active small interfering RNA (siRNA) species loaded into RISC for sequence-specific silencing of viral RNAs. Targeting NP and PA is strategic because both products are central to replication: NP supports ribonucleoprotein assembly and genome encapsidation, and PA functions as a catalytic subunit of the viral polymerase complex. Simultaneous expression of two distinct shRNAs creates multiplexed pressure across two nonredundant viral functions, which should improve robustness over a single-target design and reduce escape through mutation of one viral target (Linke et al. 2016). Thus, SiVEC-IAV combines bacterial invasion and delivery with programmable post-transcriptional specificity. Pulmonary administration produced strong antiviral activity in a murine IAV model with minimal immunogenicity and no adverse reactions (Mora et al. 2023).

SiVEC further modified SVC1 for systemic human administration and manufacturing. They introduced a mutation to mitigate lipopolysaccharide (LPS) immunogenicity (Somerville et al. 1996, 1999) and stabilized the critical invasion and phagosomal escape functions by moving *inv* and *hlyA* from the cargo-encoding plasmid to the bacterial chromosome. This new strain is commercially branded as BactPac. BactPac is currently being deployed by SiVEC as a platform to generate a multi-RAS-targeting mRNA therapeutic, SVC-KRAb. SVC-KRAb is the only system discussed here programmed to produce and deliver a eukaryote-translatable circular mRNA (mRNA^ET^). Circular RNA lacks exonuclease-sensitive 5′ and 3′ ends, potentially improving persistence over linear RNA during production, transfer, and cytosolic residence (Wang et al. 2023). Circularization is generated by a genetically encoded self-splicing pathway within the product mRNA^ET^. In this approach, the mRNA^ET^ sequence is placed between rearranged halves of a self-splicing intron (Cech 1990; Puttaraju and Been 1992) to mediate mRNA cicularization. To make this mRNA eukaryote-translatable, the construct also includes a sequence for 5′ cap-independent translational initiation, here a virus-derived internal ribosome entry site (IRES) (Gao and Wu 2025). The payload cassette is designed to encode the therapeutic open reading frame, a mechanism to generate a useful RNA topology in the bacterial cell, and the translation-initiation features required for protein production by the mammalian cell. SiVEC's ongoing pre-clinical studies show strong efficacy of SVC-KRAb both in vitro and in vivo (murine) across multiple RAS mutations and isoforms. SVC-KRAb is being developed for the treatment of mutant RAS-driven solid tumors and is intended for intravenous administration.

### *Salmonella-*based therapeutics

As a chassis organism, attenuated *S. enterica* serovar Typhimurium offers intrinsic tumor colonization, tolerability after systemic administration, and extensive genetic tools for oncology development. Research on *Salmonella*-based therapeutics has been an active area of research since 1997 (Pawelek et al. 1997), and the therapeutically directed strain VNP20009 (Low et al. 1999; Clairmont et al. 2000) was the first to reach phase 2 clinical trials (Cunningham and Nemunaitis 2001). Extensive VNP20009 development is reviewed elsewhere (Zheng and Min 2016; Liang et al. 2019; Zahedifard et al. 2025). Here we examine subsequent clinical progress. Ernest Pharmaceuticals used VNP20009 to create BacID (Raman et al. 2021, 2023). BacID illustrates two design principles: exogenous regulation of therapeutic activity and an intrinsic self-control mechanism. In VNP20009, these functions derive from elements developed by Forbes and collaborators (Raman et al. 2019, 2021, 2025) (for additional information, see also Raman et al. 2023 and references therein). BacID separates therapeutic function into genetically controlled stages. Its invasive behavior is not constitutive; instead, *flhDC*, the central regulators of flagellar synthesis, motility, and mammalian cell invasion, are regulated by the salicylate-responsive *Psal* promoter (Raman et al. 2025). This design suppresses invasion during systemic administration and induces it after tumor colonization through salicylic acid administration (the active metabolite of aspirin). In vivo, induction of *flhDC* before injection reduced tumor colonization, probably through immune recognition and premature clearance; delayed induction restored colonization and enabled invasion and cargo delivery. Thus, useful bacterial behaviors may require temporal separation, with host interaction activated only at the target site. BacID also contains an intrinsic self-limiting delivery mechanism regulated by a *PsseJ-lysE* circuit, which becomes relevant only after the bacteria have entered cancer cells. Simply increasing invasion via *flhDC* expression did not optimize delivery because it depended on vacuolar residence, where the *PsseJ-lysE* circuit is activated. While *flhDC* induction increased invasion but accelerated vacuolar escape, reducing lysis and cargo delivery. Deletion of *sseJ*, which helped retain bacteria within *Salmonella-*containing vacuoles (SCVs). In the *ΔsseJ* background, *flhDC* continued to increase invasion without reducing lysis, coupling enhanced host-cell entry (*flhDC* overexpression) with sustained vacuolar retention and intracellular release (*sseJ* deletion). These elements encode a sequence of colonization, invasion, intracellular residence, and release rather than constitutive expression. Ernest Pharmaceutical's lead therapeutic, EBT-002, appears to use the genetic architecture of BacID with an added *asd* deletion (which confers DAP auxotrophy) to deliver active cleaved caspase-3 protein, a positive effector of apoptosis. Preclinical data demonstrated that this therapeutic strain could induce cell death in pancreatic, breast, and liver cancer cells and reduce the growth of breast tumors (Raman et al. 2025).

Salspera's Saltikva (Salmonella-IL2) provides a clinically advanced example. The orally administered *S. enterica* serovar Typhimurium strain combines *cya* and *crp* attenuation with an *asd* complementation system that stabilizes a plasmid encoding C-terminally truncated human IL-2 without antibiotic selection. In a nonrandomized phase 2 study in metastatic pancreatic cancer (NCT04589234), investigators reported median progression-free and overall survival of 15 and 20.3 mo, respectively, and a 70% response rate among 20 patients receiving more than five doses with FOLFIRINOX; no serious adverse events were attributed to Saltikva. Because the analysis used a small treatment-exposed subset, included expanded-access patients, and relied on historical comparisons, the efficacy signal remains hypothesis-generating (Batist et al. 2026). An important and interesting feature of Saltikva is that the bacteria reach distal tumors after oral administration, suggesting a mechanism by which the bacteria can cross the gastrointestinal barrier to achieve systemic biodistribution.

Actym Therapeutics has also developed a *Salmonella*-based cancer therapeutic. Its “*S. Typhimurium* Anti-Cancer Therapy” (STACT) platform (Cron et al. 2025) was engineered from VNP20009 primarily to reduce immunogenicity for systemic administration. STACT retains the LPS-modifying *msbB* mutation inherited from VNP20009 as well as an additional deletion of *pagP,* which further reduces LPS detection. Flagellar expression, which can induce harmful inflammasome activation and pyroptotic cell death after flagella sensing by host Toll-like receptors 4 and 5 (TLR4, TLR5), was eliminated via *fljB* and *fliC* deletion. A deletion of *csgD*, a central biofilm regulator (Gerstel and Römling 2003), eliminated curli fimbriae, another innate immune activator detected through TLR2. They also deleted *ansB* to eliminate extracellular L-asparaginase II and increase T-cell activity, then replaced the VNP20009 Tn*10* insertion with a clean *purI.* Importantly, *purI* deletion promotes tumor enrichment and limits replication in healthy tissue. STACT cargos are encoded on plasmids under eukaryotic expression elements, with stability maintained by chromosomal *asd* deletion and plasmid-borne *asd^+^* complementation. Unlike flagellated BacID, STACT lacks flagella and is taken up by tumor-resident myeloid cells. Intracellular lysis releases plasmids that the target cells express. Actym's lead candidate, ACTM-838, delivers two immunomodulatory payloads, IL-15plex and engineered constitutively active STING (Zhang et al. 2022), to the TME after systemic administration. The cargos cooperate to activate innate and adaptive immunity, with eSTING driving inflammatory signaling and IL-15plex supporting lymphocyte activation and persistence. Preclinical data show tumor-localized delivery, tumor immune reprogramming, durable responses, and synergy with anti-PD-1 (Cron et al. 2025). In 2024, the FDA approved ACTM-838, a phase 1a/1b clinical trial (NCT06336148), which has since been terminated due to a strategic business decision, as reported by the sponsor; nevertheless, the underlying platform engineering remains a well-characterized example of an immunogenicity-reduction strategy at the chassis level.

Although both derive from VNP20009, BacID and STACT pursue different goals. BacID regulates invasion to coordinate tumor colonization, cancer-cell entry, vacuolar residence, lysis, and cytotoxic protein release. STACT suppresses immunostimulatory and nonproductive host interactions to favor systemic delivery, tumor enrichment, phagocytic uptake, and local expression of immunomodulatory cargo within the TME. Alongside VNP20009, Hoffman and colleagues developed a distinct preclinical lineage, *S. enterica* serovar Typhimurium A1-R (Hoffman 2011). The strain was generated by chemical mutagenesis for leucine and arginine auxotrophy and subsequently re-isolated from a tumor to enhance its tumor-targeting phenotype (Zhao et al. 2006). These amino acid dependencies constrain growth in normal tissues while permitting preferential proliferation in nutrient-rich tumors, enabling A1-R to produce antitumor activity without an added therapeutic payload. Although A1-R has remained preclinical, it illustrates how metabolic attenuation and in vivo selection can establish tumor selectivity at the chassis level.

### Other pathogen-derived bacteria-based therapeutics

T3 Pharma has built a therapeutic delivery platform on an attenuated *Y. enterocolitica* chassis. *Y. enterocolitica* naturally delivers protein-based cargo to eukaryotic cells via its T3SS (Davis 2018; Seabaugh and Anderson 2024). In a 2015 study, Ittig and colleagues described the rational engineering of *Y. enterocolitica* to serve as a general therapeutic protein delivery platform (Ittig et al. 2015). To this end, they first genetically modified *Y. enterocolitica* to eliminate its ability to translocate its endogenous virulence-related effectors (YopH, O, P, E, M, and T). They then deleted the aspartate-β-semialdehyde dehydrogenase gene (*Δasd*), rendering it auxotrophic for DAP and dependent on exogenous DAP supplementation for growth, a deliberate biocontainment engineering choice (functionally equivalent to the *dapE* and *dapA* deletions discussed above). In parallel, they designed plasmids optimized for high protein expression that, through standard cloning approaches, can be modified to encode cargo proteins with a translational fusion to YopE1-138, the N-terminal 138-amino-acid fragment of YopE that contains the T3S secretion signal and the SycE chaperone-binding domain but deliberately excludes the C-terminal Rho-GAP domain; thus, the fusion protein retains secretion competence while eliminating YopE's endogenous cytotoxic activity. Finally, they engineered the bacteria to overexpress the SycE chaperone, which is required for injection by the T3S apparatus (Boyd et al. 2000). The resulting platform strain could then be modified to create various therapeutics based on the relatively simple process of cloning a protein of interest into their custom expression plasmid.

Ittig and colleagues further demonstrated that iterative genetic refinement of the chassis beyond the core ΔHOPEMT/*Δasd* design could tune host-pathogen signaling interactions. Specifically, deletion of the adhesin-encoding gene *invA* reduced infection-induced host phosphorylation. This shows that additional attenuation can control off-target host signaling produced by the chassis independently of cargo. T3SS delivery imposes a constraint: substrates pass through the injectisome in an unfolded or partially folded state, leaving the YopE1-138 tag attached after translocation and potentially interfering with some payloads. Ittig and colleagues addressed this issue via two strategies. First, a TEV protease cleavage site was introduced between YopE1-138 and the cargo, and the target cells can be co-infected with a second bacterial strain delivering YopE1-138-fused TEV protease; upon translocation of both fusion proteins, the protease cleaves the tag intracellularly. Second, ubiquitin was incorporated into the fusion protein, and endogenous host deubiquitinating enzymes (DUBs) cleave it at the ubiquitin C-terminus after delivery, releasing the cargo in its native sequence without requiring any exogenous protease. The latter strategy is particularly notable from a genetic engineering standpoint, as it co-opts the host cell's own proteolytic machinery as a post-translational processing step.

T3 Pharma's lead clinical oncology asset, T3P-Y058-739 (Niu et al. 2025) (to the best of our knowledge, no study on this asset has been published; see also clinical trial NCT05120596), demonstrates a deployment of this platform. T3P-Y058-739 has a two-pronged mechanism of action in tumors: first, the presence of the bacteria, which arrive at the tumor via an intrinsic behavior of *Yersinia*, induces an anti-tumoral host immune response in the tumor microenvironment (TME); second, the T3P-Y058-739 bacteria deliver undisclosed type 1 interferon and Toll-like receptor protein payloads that induce cancer cell death.

Bacteria-based therapeutics have also been investigated in the context of immune modulation through interactions with the host immune system. To this end, Gravekamp's group has extensively explored genetic programming of a *L. monocytogenes* chassis (Chandra et al. 2013; Chandra and Gravekamp 2013; Singh et al. 2014; Gravekamp and Chandra 2015; Jahangir et al. 2017; Vitiello et al. 2019; Selvanesan et al. 2022; Lepori et al. 2024). Some outcomes of this work have been commercialized in Matter Bio's Myeloid Mediated Delivery (MMD) platform. Rather than delivering a therapeutic cargo that directly intervenes in a cellular pathway or that is itself cytotoxic, the MMD platform is designed to reprogram tumor immunogenicity. Matter Bio has deployed this platform in its AWAKE technology, which is intended to harness pre-existing tetanus-specific memory T cells generated by childhood vaccination to produce a rapid antitumor immune response (Selvanesan et al. 2022). In this approach, what they call “tetanus recall antigens” are packaged into an attenuated *L. monocytogenes*-derived vehicle that enables selective delivery to the immunosuppressed TME of solid tumors and metastases. Once localized, the bacteria invade tumor cells and deliver the antigen cargo, leading to reawakening of tetanus-specific memory T cells, thereby stimulating a strong antitumor immune response.

*Listeria* is an extremely well-suited chassis for this strategy because it brings intrinsic host-interaction traits that directly support tumor-selective delivery. The rationale for this oncology approach is that *Listeria* attracts (Chandra et al. 2013) and infects myeloid-derived suppressor cells (MDSCs) (Drevets et al. 1995), which are also recruited to primary tumors via cytokines and other attractants (Ostrand-Rosenberg and Sinha 2009; Bayne et al. 2012); thus, the MDSCs act as a co-opted delivery vehicle to bring the *Listeria* cells to the TME. Once there, the *Listeria* cells spread from the infected MDSCs into the tumor cells (Chandra et al. 2013) via an intrinsic cell-to-cell dissemination program (Dowd et al. 2020; Disson et al. 2025). While *Listeria* is rapidly eliminated from normal tissues, it can selectively enter, multiply, and persist in the immunosuppressed TME (Zhou et al. 2018); thus, the development of this system did not require the engineering of a new form of tumor tropism from scratch. Instead, it builds on a chassis with an intrinsic natural intracellular lifestyle, a mechanism of intercellular dissemination, and an ability to survive in the immune-suppressed tumor.

The underlying genetics of the engineered system build on these intrinsic chassis traits by simultaneously encoding attenuation, intracellular delivery, and cargo production. A commonly used chassis for this type of engineering is XFL7 (sometimes referred to as XFL-7) (Gunn et al. 2001). XFL7 carries a null mutation in chromosomal *prfA*, an essential positive transcriptional regulator of both itself and multiple *Listeria* virulence genes. The absence of PrfA both attenuates the strain and provides a convenient mechanism for plasmid maintenance. The therapeutic system described by Selvanesan and colleagues is built on the pGG34 plasmid, which encodes (1) a mutant *prfA* allele that partially complements the chromosomal null mutation without restoring virulence, which serves as a plasmid maintenance mechanism; and (2) the therapeutic cargo, a fusion of a noncytolytic listeriolysin O fragment (deleted for the C-terminal cholesterol-binding region and retaining vacuole escape activity) and the nontoxic tetanus toxoid (TT) fragment (TT_856-1313_), LLO-TT_856-1313_. The rationale for the translational fusion of these two elements is that LLO-mediated vacuolar escape (which is retained in the LLO fragment) was essential for intracellular delivery. This plasmid was introduced into the XFL7 strain to generate the therapeutic strain, *Listeria-*TT. When administered intraperitoneally in combination with the chemotherapeutic gemcitabine, *Listeria-*TT showed strong preclinical efficacy in two pancreatic ductal adenocarcinoma (PDAC) mouse models: an 80% reduction in tumor burden, an 87% reduction in metastases, and 40% longer survival.

### Probiotic-based therapeutics

Probiotic strains have emerged as an important class of chassis for bacteria-based therapeutics because they combine well-characterized host compatibility with varying degrees of genetic tractability. Among these, *Lactococcus* spp. and *E. coli* Nissle 1917 (EcN) illustrate distinct translational strategies. The present section focuses on Aurealis Therapeutics and Ilya as representative deployments of probiotic platforms, while EcN is discussed in greater detail below in the context of synthetic biology.

Aurealis Therapeutics has developed a live therapeutic platform on a probiotic *L. lactis* subsp. *cremoris* chassis for localized delivery of therapeutic proteins at wound sites or other therapeutically targeted tissue. *L. cremoris* is commonly used as a starter culture in cheese production (Song et al. 2017), and because of its common use in food fermentation, *L*. *lactis* has been provided generally recognized as safe (GRAS) status by the FDA. The GRAS designation provides a regulatory advantage for therapeutic products built on this chassis (Dey and Sankaran 2024); however, recombinant features do not allow assignment of simple GRAS status for derived therapeutics. A unique feature of Aurealis’ system is that a single strain can be used to simultaneously express and deliver multiple therapeutic proteins. Aurealis has used a platform strain built on a chassis of *L. cremoris* strain MG1363 (Kurkipuro et al. 2022). Their platform strain, *L. cremoris* AUC1000, is an *alr*-deleted derivative of MG1363 (Bron et al. 2002). The *alr* gene encodes an alanine racemase that catalyzes the interconversion of *D*-alanine and *L*-alanine, and loss of *alr* leads to alanine auxotrophy. This auxotrophy allows antibiotic-free plasmid maintenance via plasmid-borne *alr^+^* expression, which complements the chromosomal deletion, thereby rescuing the alanine auxotrophy. This selection system is considered food safe since there is no requirement for antibiotic use (Bron et al. 2002). This strain was also used as a platform for Aurealis’ first therapeutic asset, AUP1602-C, designed to induce tissue regeneration in chronic inflammatory wounds (Kurkipuro et al. 2022). To create the AUP-1602-C therapeutic strain, Kurkipuro and colleagues created a custom plasmid, pC-CFI. This plasmid combines the *alr* selection marker, replication functions (*repA* and *repC*), and a single operon regulated by the inducible *PgadC* promoter from the *gadCB* stress-response system, comprising codon-optimized genes for human CSF-1 (colony-stimulating factor 1), FGF-2 (fibroblast growth factor 2), and IL-4 (interleukin-4). The *PgadC* promoter is chlorine-inducible, enabling regulated expression (Sanders et al. 1997). Each of these genes is translationally fused to the *Lactococcus* Usp45 secretion signal and preceded by a defined ribosome binding site, enabling coordinated expression and secretion of all 3 proteins. The authors further showed that the therapeutic proteins were indeed produced, secreted, and correctly processed, i.e. the Usp45 signal was deleted as part of the secretion pathway (Ng and Sarkar 2013). AUP1602-C occupies a distinctive position within the systems discussed here because it replaces the more common logic of bacterial invasion, intracellular delivery, or lysis-coupled release with a probiotic, food-grade chassis engineered for coordinated secretion of 3 human therapeutic proteins after topical administration to wounds (Schindler et al. 2024). Aurealis has expanded its platform from wound healing into oncology, reporting the construction and pre-clinical data for its AUP-55 therapeutic strain (Samaranayake 2023). Unlike AUP1602, AUP-55 is administered systemically, after which the bacteria (a facultative anaerobe) traffic to the hypoxic TME (Garza-Morales et al. 2020). AUP-55 is genetically programmed to produce and deliver human IFN-α and human IL-15 from a platform plasmid, while also leveraging endogenous immunostimulatory features of the chassis itself, including surface lipoteichoic acid, which acts as a TLR2 agonist, peptidoglycan muropeptides, which act as NOD2 agonists, and CpG motifs in bacterial DNA, which act as TLR9 agonists. Through this combined architecture, AUP-55 activates both innate and adaptive antitumor immunity, suppresses angiogenesis, and promotes direct killing of cancer cells. Aurealis illustrates a distinct probiotic-based design strategy in which a food-grade probiotic chassis is engineered for multi-cargo secretion rather than invasion or lysis. Differences between AUP-1602 and AUP-55 show how activity can combine encoded cargo expression with delivery behavior and intrinsic chassis properties, with administration route determining their relative importance. Ilya Pharma's bacterial platform is based on *Limosilactobacillus reuteri* R2LC, a non-human-origin, non-colonizing lactic acid bacterial strain from a species commonly associated with probiotic use. Engineered R2LC bacteria are intended to function as transient local bioreactors that produce short-lived human proteins directly at skin or mucosal surfaces, thereby sustaining local exposure while limiting systemic distribution. In ILP100 (emilimogene sigulactibac), R2LC carries the pSIP_CXCL12-α plasmid, and addition of the activating peptide SppIP induces production and release of human CXCL12-1α. This design enables local delivery of an otherwise biochemically unstable immunomodulatory chemokine without requiring bacterial invasion or lysis. The ILP100 active substance has been developed in topical and oral formulations. ILP100-Topical is intended to promote healing of difficult wounds through local CXCL12 delivery and has been evaluated in a randomized, double-blind phase 1 study involving 240 induced wounds in 36 healthy volunteers. Treatment was well tolerated without detectable systemic exposure and shortened the time to first recorded healing by an average of 6 d, with a ten-day reduction at the highest dose (Öhnstedt et al. 2023). The topical formulation is also being developed for severe ulcerative skin lesions associated with STING-associated vasculopathy with onset in infancy. ILP100-Oral applies the same CXCL12-producing bacterial active substance to gastrointestinal delivery and is being developed as a locally acting treatment for immune checkpoint inhibitor-induced colitis. In preclinical mouse models, oral ILP100 reduced established colitis, promoted regulatory immune responses and mucosal healing, and showed no evidence of systemic exposure or bacterial engraftment (Öhnstedt et al. 2024). Ilya Pharma is also developing ILP101-Inhale, an earlier-stage candidate in which *L. reuteri* is engineered to produce CXCL17, for local treatment of pulmonary inflammation and multidrug-resistant bacterial infections in conditions including cystic fibrosis and STING-associated vasculopathy.

### Synthetic biology in bacteria-based therapeutics

Synthetic biology has enabled the development of engineered gene circuits in bacterial hosts (Brophy and Voigt 2014; Nielsen et al. 2016; Iram et al. 2024). These circuits produce condition-responsive outputs from relevant signals. Natural or engineered biosensors detect stimuli such as chemicals, temperature, and pH (Brophy and Voigt 2014; Nielsen et al. 2016; Piraner et al. 2017). In this way, bacteria can function as “smart” therapeutics in which drug release can be activated or suppressed by defined cues, improving dose control. Biosensor switches can also form more complex circuits, including logic gates (akin to the decision processors in electronic devices), that generate designed functions from combinations of inputs (Nielsen et al. 2016; Groseclose et al. 2021). Therapeutic circuits can detect disease-associated conditions such as hypoxia or altered tumor pH and trigger cargo production or release (Ozdemir et al. 2018). Well-designed circuits thus encode conditional pharmacology in living bacteria. Several companies are entering the clinic with synthetic biology-driven microbial therapeutics (e.g. Prokarium with their ZH9 *S. enterica* serovar Typhi platform), but their underlying genetic designs are not public and are therefore not discussed further.

Synlogic was an early systematic adopter of synthetic biology for live bacterial therapeutics. Synlogic's framework proceeds from problem definition through genetic design, strain construction, and deployment. Synlogic developed a platform, commercially referred to as a “Synthetic Biotic”, built on the EcN chassis. The first deployment of the Synthetic Biotic platform was in their therapeutic asset SYNB1618 (Isabella et al. 2018). This therapeutic (which was later replaced by SYNB1934 for clinical translation) is an engineered strain that can degrade intestinal phenylalanine as an alternative to dietary restriction in phenylketonuria. Unlike cargo-based examples above, SYNB1618 encodes a complete metabolic pathway to supplement the patient's physiological deficit. Synlogic constructed SYNB1618 on the EcN chassis by chromosomally inserting phenylalanine-degradation genes with elements controlling activity, stability, and containment. Three copies of *stlA*, which encodes phenylalanine ammonia lyase, and two copies of *pheP*, which encodes a high-affinity phenylalanine transporter, were integrated into the chromosome in safe harbor sequences, avoiding selective and regulatory liabilities associated with antibiotic-supported plasmid retention. To enable induction in the GI tract, these genes were placed under the anaerobically inducible *PfnrS* promoter. This promoter choice is highly relevant because it couples pathway expression to host-relevant oxygen limitation via FNR-responsive regulatory logic. Synlogic also added two additional *stlA* copies under *Ptac*, an IPTG-inducible promoter, allowing PAL induction during manufacturing so the pathway was active at dosing. The strain was engineered to carry a single copy of *pma*, encoding L-amino acid deaminase (LAAD), under the arabinose-inducible P*_BAD_* promoter, providing a second degradation route activatable before dosing with arabinose. SYNB1618 could therefore begin catabolism immediately after administration, before oxygen-sensing *fnrS* in situ, which only occurs as the bacteria enter the more distal anoxic GI environment. This design produced a stable, physiologically tuned strain that initiates catabolism at dosing and sustains activity in the gut. Another notable feature of the SYNB1618 engineering was the use of gene redundancy and genetic architecture as a mutational buffer. Multiple copies of *stlA* and *pheP* reduced the chance that one loss-of-function mutation would eliminate pathway function during scale-up. Synlogic further arranged the repeated inserts in the same chromosomal orientation and separated them by essential genes. The shared orientation biases homologous recombination toward deleting the intervening region (Lovett et al. 1994; Bi and Liu 1996; Azpiroz and Laviña 2017); because repeats flank essential genes, these events are lethal rather than yielding viable pathway-deficient recombinants. Finally, SYNB1618 was biocontained via deletion of *dapA*. SYNB1618 illustrates a genetics-forward therapeutic design logic in which pathway engineering, promoter choice, chromosomal architecture, mutation tolerance, and biocontainment are interdependent properties. Together, these choices stabilize the therapeutic, confer environmental responsiveness, and limit escape variants, especially during large-scale manufacturing (Rugbjerg et al. 2018; Mu and Zhang 2023). Synlogic has used a similar design logic in generating additional clinical-stage pipeline assets, i.e. SYNB1934, a further optimized derivative of SYNB1618 (Kalantari et al. 2023); SYNB1353 (Perreault et al. 2024), for classical homocystinuria (HCU); and SYNB8802 (Lubkowicz et al. 2022; Kalantari et al. 2023) for enteric hyperoxaluria. SYNB1934 proceeded into a phase 3 clinical trial (NCT05764239, Synpheny-3); unfortunately, this trial was terminated in 2024 because it was unlikely to meet its primary endpoint.

A complementary example of synthetic biology in bacteria-based therapeutics is provided by GenCirq's synchronized lysis circuit (SLC) (Din et al. 2016). The reusable SLC couples positive and negative feedback to oscillate the bacterial population and cycle payload production and release. Its quorum-coupled program uses the LuxI/LuxR signaling module (Fuqua et al. 1994; Soto-Aceves et al. 2023) and the bacteriophage *ϕ*X174 E lysis gene (Witte and Lubitz 1989; Witte et al. 1990). The *luxI* promoter controls the level of an autoinducer, acyl-homoserine lactone (AHL) (Galloway et al. 2011). AHL then binds to the bacterially expressed protein LuxR, which then further induces transcription from the *luxI* promoter. The *ϕ*X174 E gene is also controlled by the *luxI* promoter, so they too respond to the levels of LuxR-AHL. Because LuxR-AHL diffuses to neighboring cells, the population can sense density. At a threshold, *ϕ*X174 E triggers coordinated lysis, releasing intracellular payload and reducing the population. Surviving bacteria restart the cycle. To demonstrate therapeutic function in vivo, Din et al. (2016) placed the SLC in a *S. enterica* serovar Typhimurium chassis and then introduced *E. coli hlyE*, which encodes cytotoxic hemolysin E, as the cargo. *HlyE* was also placed under the control of the *luxI* promoter such that its expression was also controlled by the LuxR-AHL level. In mice with subcutaneous colorectal tumors, the strain produced intratumoral population oscillations and an antitumor effect. It was also effective in a mouse model of colorectal liver metastasis and delivered previously developed cargos, establishing the SLC as reusable when compatible with the mechanism. The circuit applies when bacteria proliferate in patients and release cargo extracellularly, not when they must enter target cells. It avoids payload preloading and engineered secretion machinery while reducing the risk of systemic inflammation.

### Liabilities associated with genetic engineering in bacteria-based therapeutics

Liabilities in bacteria-based therapeutics arise from genetic and phenotypic instabilities and potential failure of the biological properties of the chassis and its evolutionary behavior to support therapeutic goals. This section addresses chassis liabilities, including targeting and persistence, and genetic liabilities created by selection during manufacturing and in vivo use (Fig. 3). Engineering bacteria for therapeutic use is an exercise in spatial and temporal control that ensures that concentrated activity is maintained at a disease site for as long as needed but terminated before it causes harm. For example, colitis in mice was reduced by 50% when *L. lactis* delivered IL-10 directly to the gut mucosa via intragastric administration, whereas systemic administration was less effective (Steidler et al. 2000). However, an early clinical trial of attenuated *S. typhimurium* showed that tumor tropism alone was insufficient, as colonization at the highest tolerated dose was accompanied by thrombocytopenia, persistent bacteremia, and no tumor regression (Toso et al. 2002). Together, these results show that localization, persistence, and host-cell interaction require simultaneous design. Each can be both a design objective and a liability arising from chassis biology.

**Fig. 3.**
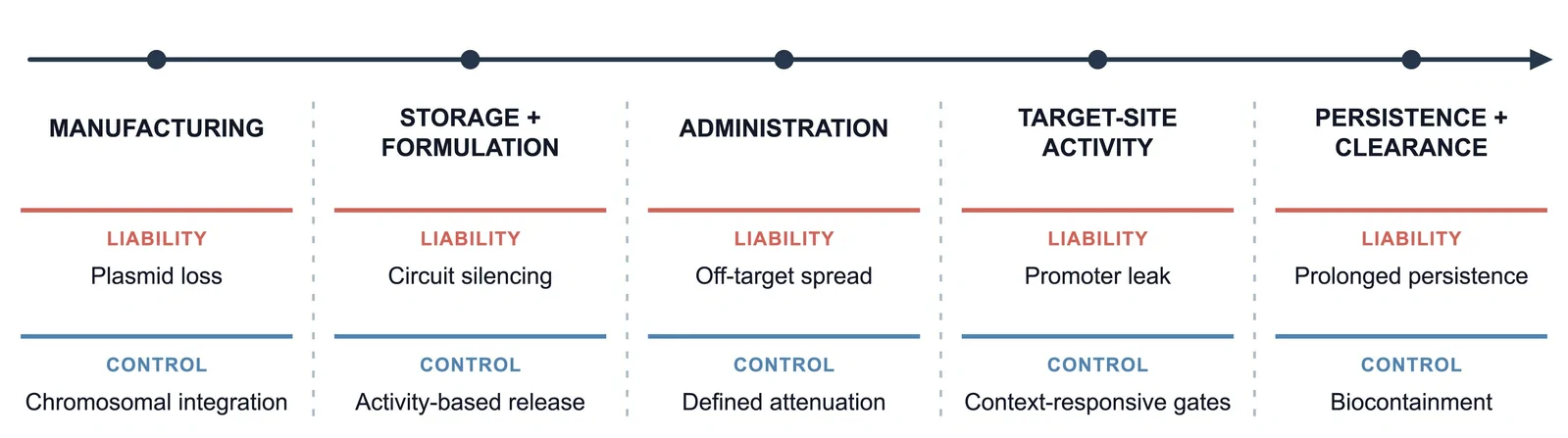
Translational liabilities and engineering controls across the product lifecycle of engineered bacterial therapeutics. During manufacturing, expression burden, plasmid loss, recombination, and escape subpopulations can arise during bacterial expansion; relevant controls include chromosomal integration, antibiotic-free selection, whole-genome sequencing, and activity-based potency assays. During storage and formulation, physiological-state drift, promoter or circuit silencing, state-transition failure, and divergence between viability and activity can be addressed through defined induction states, dosing-state release testing, transcriptional profiling, and standardized induction protocols. At administration, functional-dose variation, off-target biodistribution, innate immune activation, and premature clearance motivate measurement of colony-forming units together with activity-based potency, genetically defined attenuation, reduced immunogenicity, and biodistribution testing. At the target site, promoter leak, incomplete activation, cargo-stoichiometry errors, and insufficient localization can be controlled through promoter-specificity testing, context-responsive gates, staged genetic control, and balanced cargo stoichiometry. After the therapeutic window closes, prolonged persistence, systemic spread, continued immune stimulation, and incomplete clearance can be mitigated through exogenous-factor dependency, essential-gene repression, synthetic amino-acid dependency, and inducible capsule control.

Microbial therapeutics require concentrated activity at the disease site while sparing healthy tissue, making localization central to efficacy and safety. Some hosts provide selectivity through intrinsic tissue preferences. Facultative anaerobes, e.g. *Salmonella*, naturally accumulate within tumor tissue because the hypoxic tumor core resembles an evolved niche (Silva-Valenzuela et al. 2016); however, the same regulatory, metabolic, and stress-response networks might support survival in unintended compartments with similar features. Passive enrichment lacks precision when inflammation, necrosis, or hypoxia also occurs outside the target. Even engineered pH- or oxygen-responsive circuits and AND-gate logic that limit off-target colonization (Chien et al. 2022) remain constrained by chassis physiology, promoter behavior, signal specificity, regulatory leak, and environmental overlap.

Persistence is a second major chassis liability. Duration of activity affects cargo production, dosing frequency, host exposure, and the ability to terminate therapy after adverse events. Some chassis clear soon after dosing stops. In healthy subjects receiving oral *E. coli* Nissle for 28 d, all subjects cleared the strain after dosing ended, with a median clearance of approximately 1 wk, driven by colonization resistance from the resident microbiota (Kurtz et al. 2018). While this type of transient colonization is inherently self-limiting, it might require repeated dosing to maintain therapeutic cargo levels, a practical hurdle that has contributed to mixed outcomes in early clinical trials (Toso et al. 2002; Kurtz et al. 2018). Prolonged persistence also increases opportunities for systemic spread, immune stimulation, or unnecessary chronic exposure. Persistence is both a clinical outcome and a composite genotype-dependent phenotype involving nutrient use, stress tolerance, immune susceptibility, colonization factors, and microbiome competition. Controllable persistence requires deliberate engineering. Coupling bacterial survival to an exogenous factor through engineered auxotrophy, essential gene repression, or synthetic amino acid dependency can convert persistence into a dosing parameter (Mandell et al. 2015; Stirling et al. 2017). Inducible capsular polysaccharides can transiently protect bacteria during systemic delivery and then permit host clearance, increasing the maximum tolerated dose tenfold in murine models (Harimoto et al. 2022). Chronic applications instead require chassis and cargo designed for stable long-term colonization. Chassis liabilities arise because useful native traits evolved outside clinical constraints. Tropism, environmental responsiveness, persistence, and host-cell engagement can be harnessed therapeutically, but none is inherently benign or separable from genotype.

Therapeutic engineering also reshapes selection during manufacturing, storage, and administration. Genetic instability can arise through compensatory mutation, promoter silencing, plasmid loss, rearrangement, copy-number variation, or enrichment of non-effective subpopulations that escape physiological or genetic burden and gain a growth advantage (Foster 1993; Quiñones-Soto and Roth 2011; Williams 2016), or from a known feature of the genetic background. Genetic durability therefore asks whether engineered features remain coupled to therapeutic behavior during replication, especially in manufacturing, and under competitive or selective forces. The following examples show liabilities at multiple levels.

The detailed study of tumor-targeting *Salmonella* shows that intended modifications alone may not optimize a chassis. VNP20009 was developed as a stable attenuated strain whose defining modifications remained stable during preclinical and early clinical development (Clairmont et al. 2000); however, later work showed that the strain also carried an unintended *cheY* mutation that impaired chemotaxis (Broadway et al. 2015). A chassis can therefore retain its intended genotype while harboring background lesions that limit performance. Complementation of the mutant *cheY* allele with the wild-type gene only partly restored chemotaxis, indicating broader limitations in the genomic background (Broadway et al. 2015). Therapeutic strains therefore require whole-genome evaluation and sequencing during development and manufacturing. The *cheY* example shows that hidden limitations may appear only under conditions relevant to therapeutic function, which preclinical qualification must reproduce.

Plasmid-based therapeutics face loss, segregation instability, structural rearrangement, and copy-number variation (Summers and Sherratt 1984; Friehs 2004; Hernandez-Beltran et al. 2024) that can separate therapeutic or safety functions from an otherwise intact chassis. Moving key functions to the chromosome can mitigate this risk (Saleski et al. 2021). Development of BactPac suggests that invariant functions such as invasion and phagosomal escape belong on the chromosome, while indication-specific payloads can remain plasmid-borne. The core-modular boundary must be established empirically for each chassis and therapeutic context.

Strong selection can generate divergent subpopulations in systems using environmentally responsive promoters or other state-dependent regulation. For example, mutations conferring bacteriophage resistance are well known (Hyman and Abedon 2010). Do the persisting bacteria in the SLC-expressing population accumulate unknown mutations that alleviate the selective pressure imposed by expression of the *ϕ*X174 E lysis gene? The same concern applies to any system under strong selection.

A related clinical translational liability is that potency depends on both genotype or nominal dose and the physiological state at administration. Manufacturing, induction, formulation, and storage can alter circuit expression, stress physiology, surface state, and metabolic activity, changing readiness to execute the program in vivo (Mu and Zhang 2023). This is especially important for environmentally responsive control. SYNB1618, for example, divides pathway activity between pre-dosing induction and host-triggered activation: P*tac*- and PBAD-dependent induction can launch phenylalanine-catabolic capacity before administration, while the anaerobically inducible *PfnrS* promoter couples full pathway expression to intestinal oxygen limitation. Potency therefore depends on sequence and preservation of cells poised to transition between encoded states at dosing. Two lots with the same sequence and dosing metric, such as colony-forming units (CFUs), might therefore differ substantially in functional competence at administration. Sequence confirmation and viable count can overestimate comparability when they omit genotype-dependent physiological state. A fuller framework might combine functional assays at dosing, transcriptional profiling of circuit state, and standardized induction in release criteria. The field has not converged on such a framework, leaving an underappreciated translational gap.

Reproducible manufacturing and clinical translation depend on genomic stability that couples engineered features to therapeutic behavior. These considerations underscore the rigorous engineering standards required for bacteria-based therapeutics.

### The next frontier: bacterial delivery of gene editing systems

Delivery of gene-editing systems concentrates many design problems in one platform. Although nucleases, guides, and repair strategies are available, delivery to the correct cells with adequate precision, efficiency, and timing remains the main bottleneck. Unlike preloaded nonliving vehicles, bacteria can respond to environmental or external stimuli. A single strain can therefore integrate component production, tissue and cell targeting, sensing, controlled persistence, and staged deployment.

The principal challenge is organizing and expressing the editing components. Cargo may be DNA, RNA, protein, or a multicomponent assembly (i.e. ribonucleoproteins, or RNPs), each imposing different architectural constraints. A nuclease, guide RNA, and, when needed, repair template must reach the same cell and nucleus at suitable stoichiometry and timing. Delivery therefore requires coordinated bacterial behavior, intracellular transfer, cargo stability, and regulated activity.

Promoter selection matters because editing machinery can burden the bacterial vehicle. Large nucleases such as Cas9 and high guide RNA expression can cause off-target bacterial genome cutting (Cui and Bikard 2016), altered growth and morphology (Lee et al. 2016; Cho et al. 2018), or select variants with reduced editor expression. In multicomponent systems, promoter architecture also controls stoichiometry (Ponchon et al. 2013). Synthetic bacterial promoter libraries, including the collection developed by Anderson and others (Wang et al. 2020), can streamline promoter identification and testing.

Chromosomal integration offers genetic stability and predictable copy number (Mairhofer et al. 2013; Saleski et al. 2021), whereas plasmids provide flexibility and rapid platform modification but introduce copy-number variation and sequence instability (Summers and Sherratt 1984), segregation instability (Friehs 2004), and heterogeneous expression levels across bacterial cells (Hernandez-Beltran et al. 2024). In editing systems, loss or imbalance of one component can disable the system. A donor repair template for homology-directed repair in precision gene editing adds complexity. The donor may share a plasmid with the nuclease and guide or occupy a second plasmid. It must reach the nucleus before or near nuclease activity, requiring coordination of nucleic-acid and protein delivery and nuclear import. Conventional localization signals can import the nuclease, but repair sequences require approaches such as DNA nuclear targeting sequences, especially in non-dividing cells (van Gaal et al. 2011; Bai et al. 2017; Le Guen et al. 2021). Dividing cells simplify delivery because nuclear envelope breakdown permits donor entry.

These constraints differ between ex vivo editing, performed in cells removed from and returned to the patient, and in vivo editing. Ex vivo applications allow direct control of dose, exposure, intracellular transfer, and vehicle removal (Li et al. 2020; Almeida-Oliveira et al. 2025), making them more tractable clinically. In vivo editing requires precise targeting and mitigation of permanent off-target edits (Raguram et al. 2022; Wang et al. 2026). Bacterial delivery also increases risks of construct dissemination, prolonged delivery, vehicle persistence, and incomplete intracellular control. These challenges may exceed those of conventional bacterial cargos, although lower manufacturing costs could offset them.

SiVEC Biotechnologies is developing its BactPac platform for the simultaneous production and intracellular delivery of a gene-editing nuclease, a single guide RNA, and a repair template within a single bacterial strain. After optimization of promoter choice, coding sites, and gene copy number, studies have shown production of active RNPs and delivery in vitro. This program will test whether an engineered bacterial platform can produce and deliver a clinical gene-editing therapeutic. Ongoing studies are evaluating whether this approach can achieve functional gene editing and support development of BactPac as a clinically translatable gene editing platform.

## Conclusion

Bacterial therapeutics are genetically programmed medicines whose translation depends on precise control of microbial biology. The programs reviewed here integrate chassis selection, attenuation, payload architecture, and regulatory logic. Early studies established cargo production and transfer; clinical and near-clinical programs must also maintain engineered behavior under selection, control biodistribution and persistence, and preserve genotype-to-phenotype relationships from manufacturing through administration.

Across platforms, performance depends less on payload sophistication than on organizing localization, host-cell engagement, persistence, and immune interaction for therapeutic function. Likely translational failures include off-target biodistribution, loss of engineered function under selection, and manufacturing-induced changes in the physiological state required in vivo. Genetic durability and genotype-to-phenotype fidelity are therefore as important as payload design. The field still needs a qualification framework for both.

Complete gene-editing systems are the most demanding synthesis of these principles, requiring coordinated genetic architecture, stoichiometry, intracellular trafficking, and nuclear import in a genetically stable, manufacturable, and sufficiently attenuated system acceptable to regulators. Progress requires integrated design and systematic control of stability and administration-state fidelity. The ultimate question is whether the genetic systems that encode this capability can be designed to be precise, stable, and reproducible enough to function as reliable therapeutics.
